# Supplementary material for: Promiscuous molecules for smarter file operations in DNA-based data storage
Source: Nat Commun. 2021 Jun 10;12:3518. doi: 10.1038/s41467-021-23669-w (PMC8192770; doi:10.1038/s41467-021-23669-w)
Supplement: Supplementary file 1 — Supplementary Information [file 41467_2021_23669_MOESM1_ESM.pdf]

# Supplementary Figures

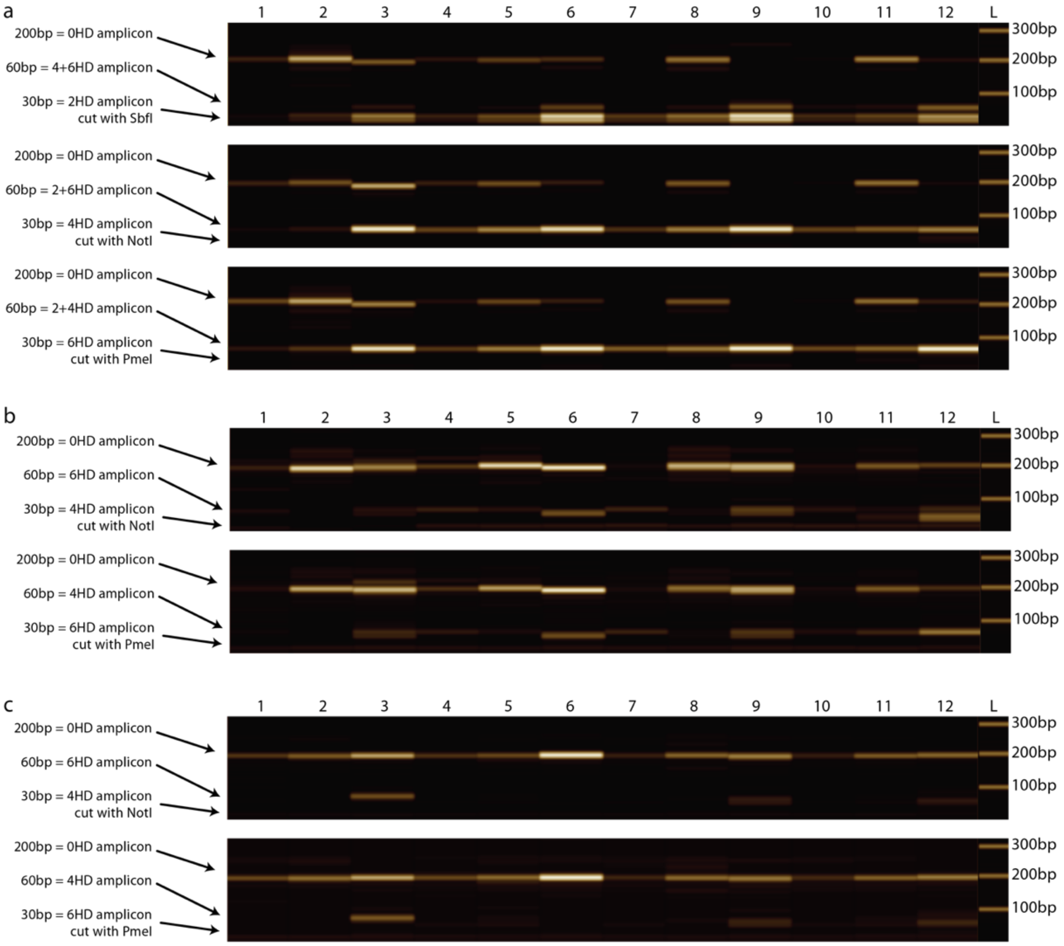


**Supplementary Figure 1. Capillary DNA gel electrophoresis of different Hamming distance PCR products derived from a screen of temperature and primer conditions.** (a) 0, 2, 4, and 6 HD or (b, c) 0, 4, and 6 HD strands were combined and amplified at the various conditions described below. Full length PCR products were 200 bp for 0HD strands and 60 bp for 2, 4, and 6 HD strands. 0 HD amplicons were left uncut while 2 HD strands were cut only by SbfI, 4 HD amplicons were cut only by NotI, and 6HD amplicons were cut only by PmeI. Gel lane numbers correspond to the following qPCR primer and annealing temperature descriptions: 1 – 125 nM primer, 60°C; 2 – 250 nM primer, 60°C; 3 – 500 nM primer, 60°C; 4 – 125 nM primer, 55°C; 5 – 250 nM primer, 55°C; 6 – 500 nM primer, 55°C; 7 – 125 nM primer, 50°C; 8 – 250 nM primer, 50°C; 9 – 500 nM primer, 50°C; 10 – 125 nM primer, 40°C; 11 – 250 nM primer, 40°C; 12 – 500 nM primer, 40°C. L equals ladder. Each experiment was run a single time. Source data are provided as a Source Data file.

a

| Rxn # | Annealing Temp | [Primer] | Anneal/Ext Time | Cycles | Taq Polymerase | [dNTPs] | MgCl2 | KCl (in buffer) | BSA | Triton X-100 | Tween 20 | DMSO | Betaine |
|---|---|---|---|---|---|---|---|---|---|---|---|---|---|
| 1 | 45C | 500mM | 30/30s | 30 cycles | 1.25U | 0.2 mM | 1.5mM | 1x | - | - | - | - | - |
| 2 | 40C | 500mM | 30/30s | 30 cycles | 1.25U | 0.2 mM | 1.5mM | 1x | - | - | - | - | - |
| 3 | 45C | 1000nM | 30/30s | 30 cycles | 1.25U | 0.2 mM | 1.5mM | 1x | - | - | - | - | - |
| 4 | 45C | 250nM | 30/30s | 30 cycles | 1.25U | 0.2 mM | 1.5mM | 1x | - | - | - | - | - |
| 5 | 45C | 500mM | 20s/20s | 30 cycles | 1.25U | 0.2 mM | 1.5mM | 1x | - | - | - | - | - |
| 6 | 45C | 500mM | 60s/60s | 30 cycles | 1.25U | 0.2 mM | 1.5mM | 1x | - | - | - | - | - |
| 7 | 45C | 500mM | 30/30s | 25 cycles | 1.25U | 0.2 mM | 1.5mM | 1x | - | - | - | - | - |
| 8 | 45C | 500mM | 30/30s | 40 cycles | 1.25U | 0.2 mM | 1.5mM | 1x | - | - | - | - | - |
| 9 | 45C | 500mM | 30/30s | 30 cycles | 0.625U | 0.2 mM | 1.5mM | 1x | - | - | - | - | - |
| 10 | 45C | 500mM | 30/30s | 30 cycles | 2.5U | 0.2 mM | 1.5mM | 1x | - | - | - | - | - |
| 11 | 45C | 500mM | 30/30s | 30 cycles | 1.25U | 0.8 mM | 1.5mM | 1x | - | - | - | - | - |
| 12 | 45C | 500mM | 30/30s | 30 cycles | 1.25U | 0.2 mM | 0.75mM | 1x | - | - | - | - | - |
| 13 | 45C | 500mM | 30/30s | 30 cycles | 1.25U | 0.2 mM | 3mM | 1x | - | - | - | - | - |
| 14 | 45C | 500mM | 30/30s | 30 cycles | 1.25U | 0.2 mM | 1.5mM | 2x buffer | - | - | - | - | - |
| 15 | 45C | 500mM | 30/30s | 30 cycles | 1.25U | 0.2 mM | 1.5mM | 1x | 0.10% | - | - | - | - |
| 16 | 45C | 500mM | 30/30s | 30 cycles | 1.25U | 0.2 mM | 1.5mM | 1x | 1% | - | - | - | - |
| 17 | 45C | 500mM | 30/30s | 30 cycles | 1.25U | 0.2 mM | 1.5mM | 1x | - | 0.10% | - | - | - |
| 18 | 45C | 500mM | 30/30s | 30 cycles | 1.25U | 0.2 mM | 1.5mM | 1x | - | 1% | - | - | - |
| 19 | 45C | 500mM | 30/30s | 30 cycles | 1.25U | 0.2 mM | 1.5mM | 1x | - | - | 0.10% | - | - |
| 20 | 45C | 500mM | 30/30s | 30 cycles | 1.25U | 0.2 mM | 1.5mM | 1x | - | - | 1% | - | - |
| 21 | 45C | 500mM | 30/30s | 30 cycles | 1.25U | 0.2 mM | 1.5mM | 1x | - | - | - | 2% | - |
| 22 | 45C | 500mM | 30/30s | 30 cycles | 1.25U | 0.2 mM | 1.5mM | 1x | - | - | - | 8% | - |
| 23 | 45C | 500mM | 30/30s | 30 cycles | 1.25U | 0.2 mM | 1.5mM | 1x | - | - | - | - | 0.1 mM |
| 24 | 45C | 500mM | 30/30s | 30 cycles | 1.25U | 0.2 mM | 1.5mM | 1x | - | - | - | - | 3.5mM |
| 25 | 45C | 500mM | 30/30s | 30 cycles | 1.25U | 0.2 mM | 1.5mM | 1x | - | - | - | 2% | 0.1mM |

b

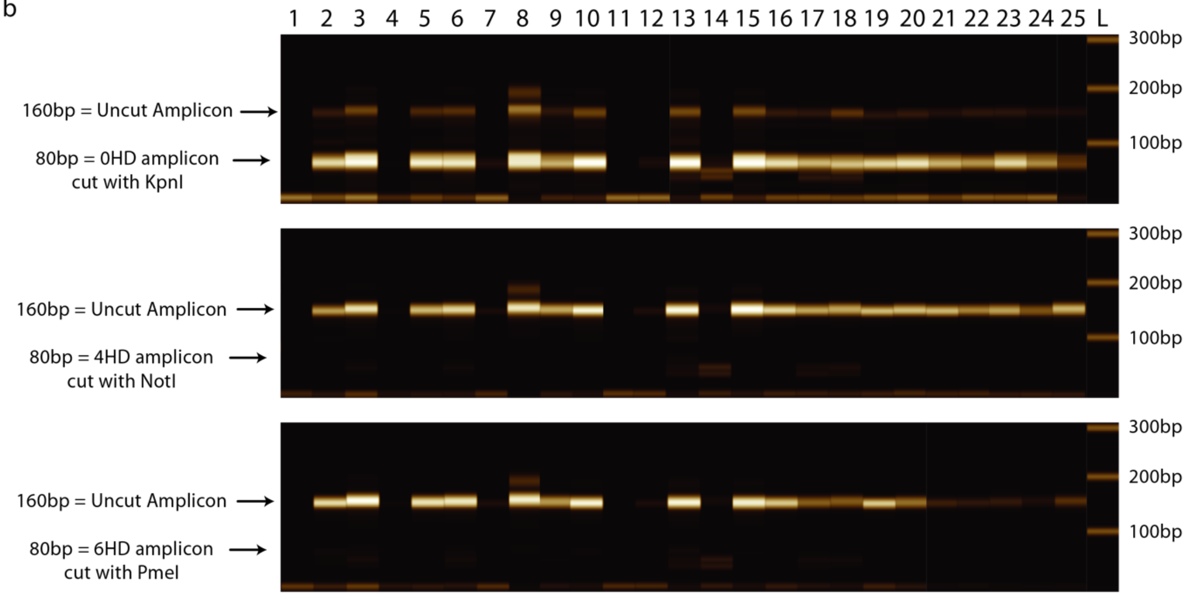


**Supplementary Figure 2. Description of a preliminary PCR condition screen for full file access.** (a) This table shows the reaction number to reference in panel b, annealing temperatures, primer concentrations, annealing and extension times, cycles counts, polymerase concentration, dNTP concentration, $MgCl_2$ concentration, KCl concentration, % BSA, % Triton X-100, % Tween20, % DMSO, and % Betaine. (b) Capillary DNA gel electrophoresis. Wright Glider 2 (0, 4, and 6 HD) strands were combined and amplified at the various reaction conditions (#1-25) described in panel a. Full length PCR products were 160 bp for all strands. 0 HD amplicons were cut only by KpnI, 4 HD amplicons were cut only by NotI, and 6HD amplicons were cut only by PmeI. Each experiment was run a single time. Source data are provided as a Source Data file.

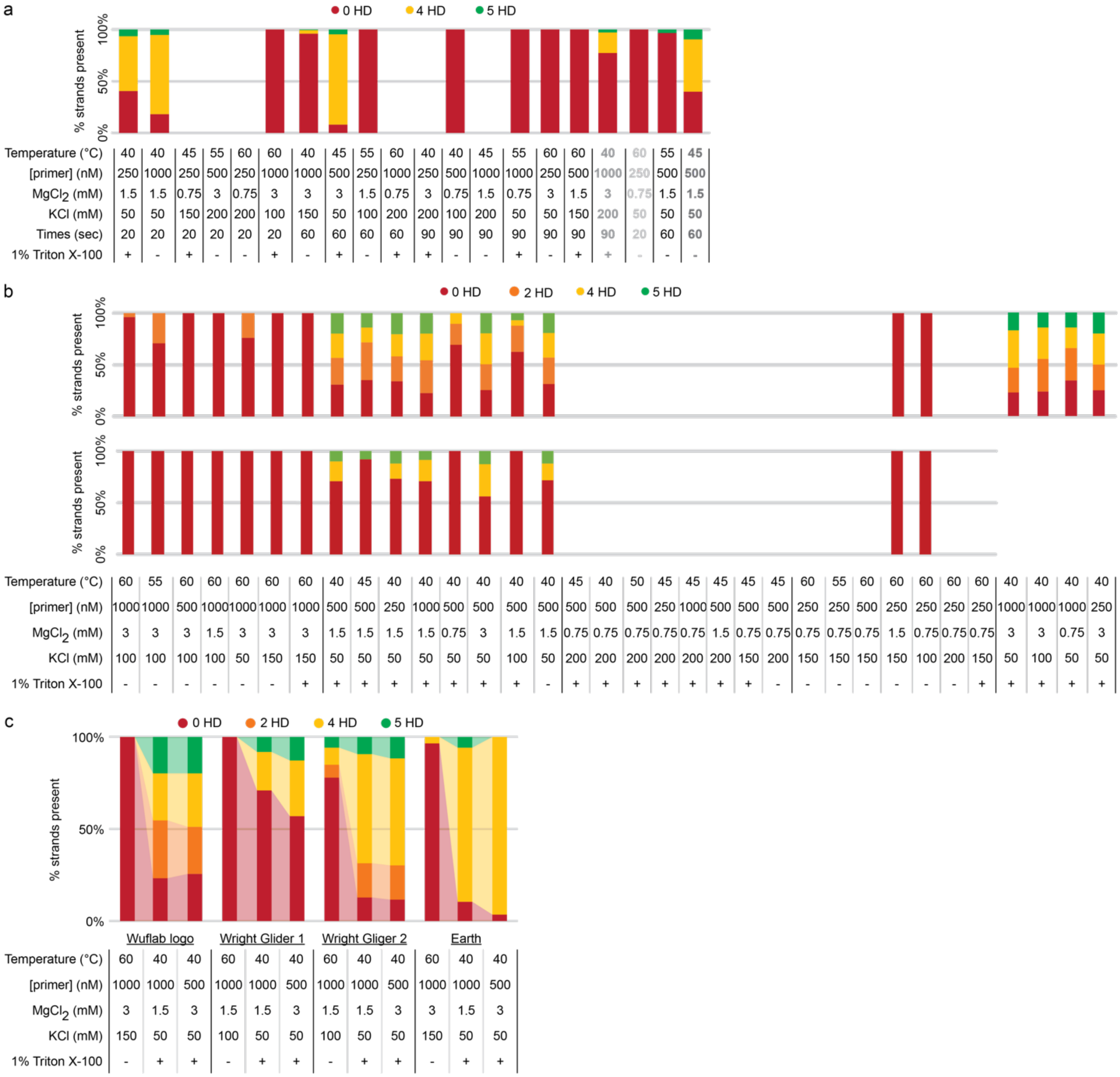


**Supplementary Figure 3. Assessing the effects of specific environmental conditions on File Preview.** (a) PCR condition screen amplifying Wright Glider 1 using relevant parameters determined in Supplementary Figure 2. (b) Fine-tuned (single variable changed per reaction) screen of PCR conditions amplifying Wuflab logo (top row) and Wright Glider 1 (bottom row). The four rightmost conditions of the top row include an attempt to find synergistic variable conditions. (c) Capillary electrophoresis analysis of Wuflab logo, Wright Glider 1, Wright Glider 2, and Earth samples which were accessed, then sent for NGS and decoded. Source data are provided as a Source Data file.

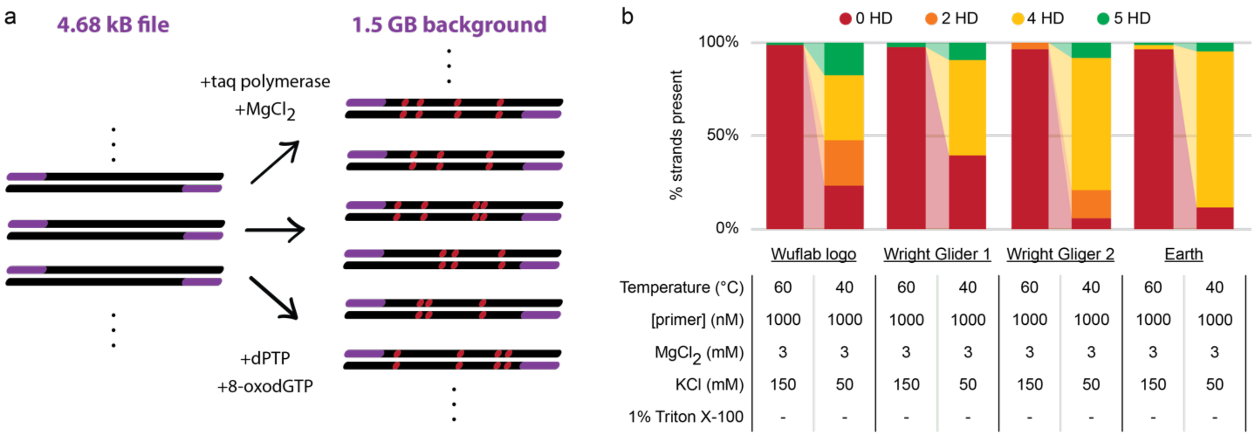


**Supplementary Figure 4. File Preview amongst a high background of data.** (a) Schematic of Error Prone PCR used to generate a 1.5 GB background by mutagenizing a file that encodes the Declaration of Independence. (b) Capillary electrophoresis analysis of Wuflab logo, Wright Glider 1, Wright Glider 2, and Earth samples which were accessed in the presence of the error prone background, then sent for NGS and decoded. Source data are provided as a Source Data file.

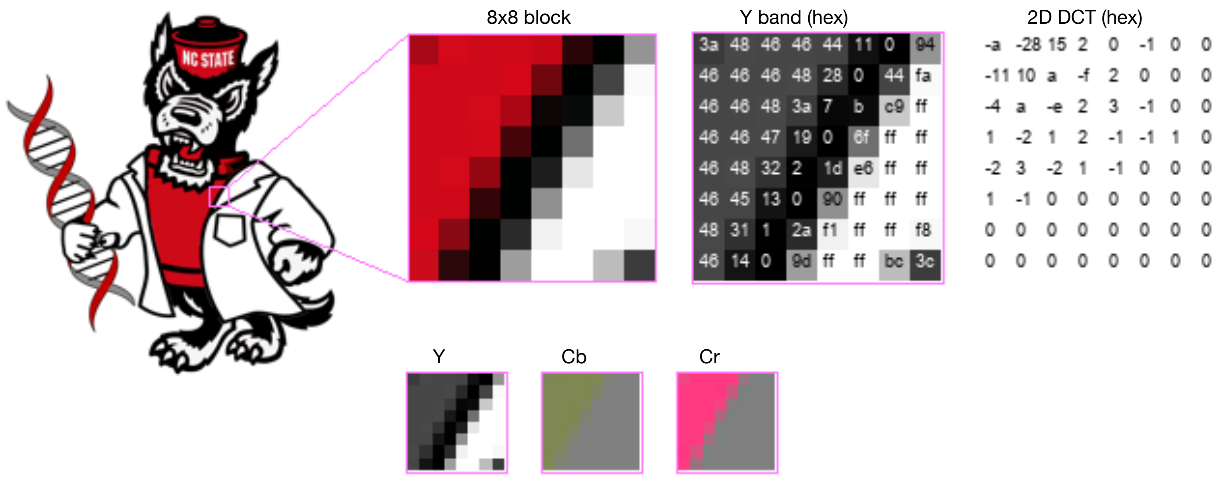


**Supplementary Figure 5. WufLab logo with an 8x8 block extracted and magnified.** The Y, Cb, and Cr bands are shown individually across the bottom row. The Y band values are shown in hexadecimal in the top middle. The top right shows the 2D DCT transformation in hexadecimal on the 8x8 block. Note the prevalence of 0 values in the bottom right corner enable significant compression.

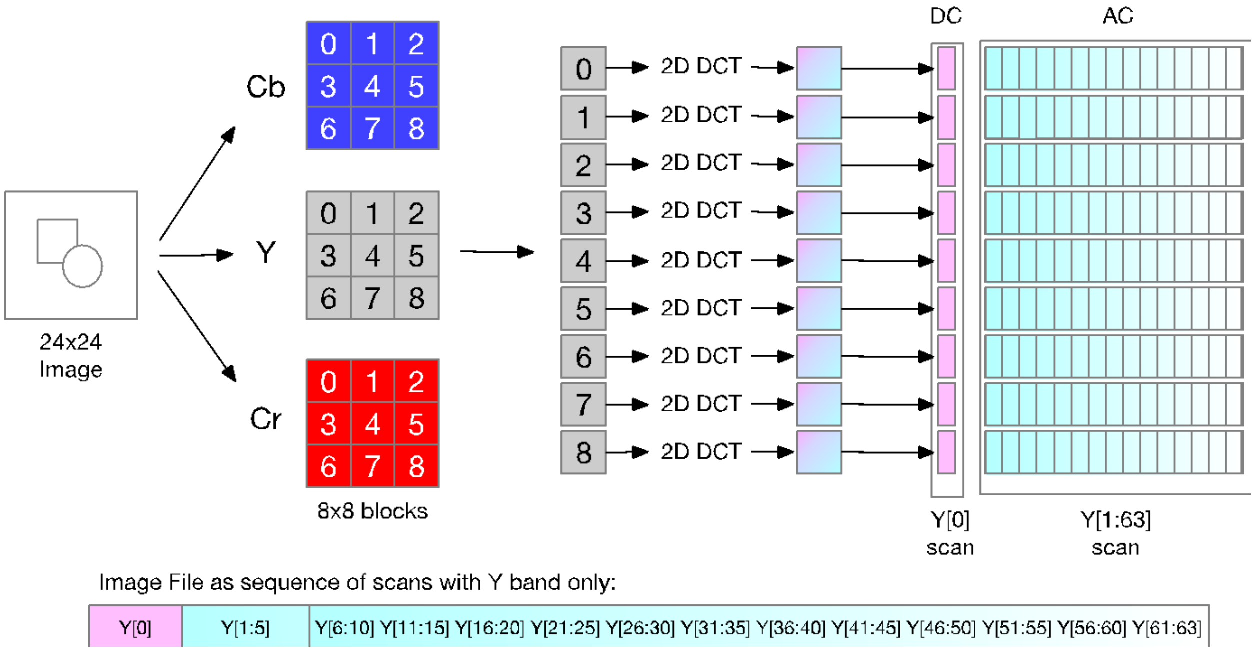


**Supplementary Figure 6. An illustration of progressive encoding for JPEG images.** A 24x24 is split into the Y, Cb, and Cr bands and divided into nine 8x8 blocks. The Y band is further shown with each 8x8 block converted into the frequency domain using the 2D DCT. The DC component across all blocks are collected into the Y[0] scan and the remaining AC components follow it in subsequent scans.

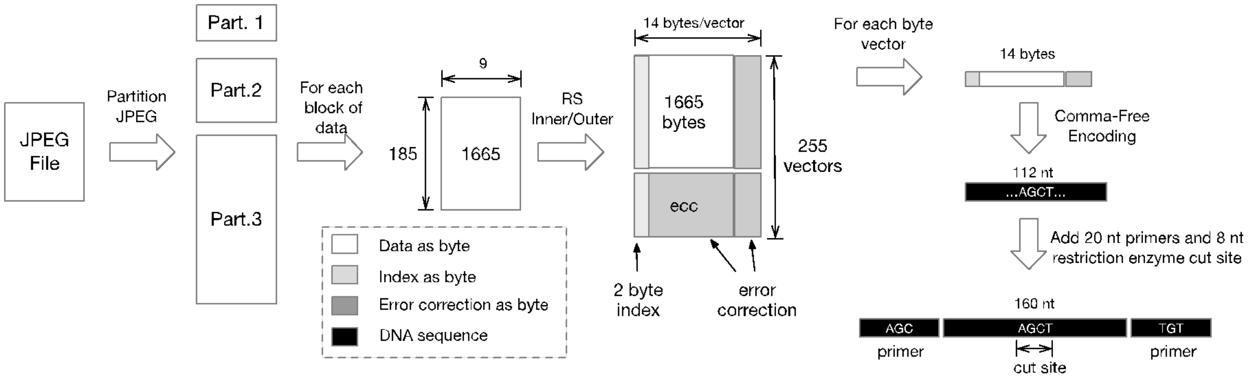


**Supplementary Figure 7. Encoding process from JPEG file into DNA.**

# Supplementary Tables

**Supplementary Table 1. Additional screening of 30 variable binding sites.** This table shows primer sequence information, original primer homology of the variable binding site, Hamming distance of the binding site to the original primer, percentage of the 30 strands amplified at constant annealing temperature while varying primer concentration (same calculation as Figure 1b, d, and e), percentage of the 30 strands amplified at constant primer concentration and varying annealing temperature, and percentage of the 30 strands that were amplified at all 4 of these conditions.

| Original primer 1 sequence: CAGGTACGCAGTTAGCACTC | | | | Original primer 1' sequence: CGTGGCAATATGACTACGGA | | | |
|---|---|---|---|---|---|---|---|
| Original Primer Homology | Hamming Distance | Variable binding sites tested | 55°C | | 250nM | | Amplified at all conditions |
| | | | 125nM | 500nM | 40°C | 60°C | |
| 1 / 1' | 2 | 30 | 90% | 100% | 53% | 77% | 43% |
| 1 / 1' | 4 | 30 | 7% | 77% | 10% | 10% | 0% |
| 1 / 1' | 6 | 30 | 0% | 67% | 10% | 7% | 0% |

**Supplementary Table 2. Competitive screening of three additional primer sequences.** This table shows original primer sequences, homology, Hamming distance of the binding site to the original primer, ratio of mismatch to perfect match strands at constant annealing temperature while varying primer concentration and each strand's tunability (same calculation as Figure 2b), and ratio of mismatch to perfect match strands at constant primer concentration while varying annealing temperature and each strand's tunability.

| | |
|---|---|
| Original primer 2 sequence:<br>CAGGAGAATGCCTTCCTAGG | Original primer 2' sequence:<br>CCTCGGTTCTTCTTGACCAG |
| Original primer 3 sequence:<br>AGGCTGGAGGTCCAATCTTG | Original primer 3' sequence:<br>ATTCTGGCCACTTCCTGAAG |
| Original primer 4 sequence:<br>AACTAAACGGAGGCCAACAG | Original primer 4' sequence:<br>TTTGTCCAGGAGCCTTTGAG |

| Original Primer Homology | Hamming Distance | 55°C | | | 250nM | | |
|---|---|---|---|---|---|---|---|
| | | 125nM | 500nM | Tunability | 40°C | 60°C | Tunability |
| 2 / 2' | 2 | 0.03 | 0.22 | 0.20 | 10.30 | 0.24 | 10.06 |
| 2 / 2' | 2 | 1.79 | 0.06 | -1.73 | 6.69 | 0.38 | 6.31 |
| 2 / 2' | 2 | 0.06 | 0.16 | 0.09 | 6.05 | 0.79 | 5.26 |
| 2 / 2' | 2 | 0.06 | 0.15 | 0.09 | 4.22 | 0.48 | 3.74 |
| 2 / 2' | 2 | 0.01 | 0.02 | 0.02 | 10.97 | 0.25 | 10.72 |
| 2 / 2' | 3 | 0.21 | 0.00 | -0.21 | 0.49 | 0.00 | 0.49 |
| 2 / 2' | 3 | 0.39 | 0.00 | -0.39 | 0.46 | 0.56 | -0.10 |
| 2 / 2' | 3 | 0.04 | 0.00 | -0.04 | 3.58 | 0.09 | 3.48 |
| 2 / 2' | 3 | 0.05 | 0.00 | -0.05 | 0.00 | 0.14 | -0.14 |
| 2 / 2' | 3 | 0.01 | 0.00 | -0.01 | 0.00 | 0.00 | 0.00 |
| 3 / 3' | 2 | 0.12 | 0.83 | 0.71 | 4.79 | 0.04 | 4.75 |
| 3 / 3' | 2 | 0.00 | 0.10 | 0.10 | 1.64 | 0.00 | 1.63 |
| 3 / 3' | 2 | 0.01 | 0.40 | 0.39 | 6.68 | 0.47 | 6.20 |
| 3 / 3' | 2 | 10.88 | 0.30 | -10.59 | 7.08 | 0.49 | 6.59 |
| 3 / 3' | 2 | 0.07 | 0.54 | 0.48 | 4.45 | 0.04 | 4.41 |
| 3 / 3' | 3 | 0.00 | 0.00 | 0.00 | 0.01 | 0.00 | 0.01 |
| 3 / 3' | 3 | 0.00 | 0.00 | 0.00 | 0.14 | 0.00 | 0.14 |
| 3 / 3' | 3 | 0.00 | 0.00 | 0.00 | 0.02 | 0.00 | 0.02 |
| 3 / 3' | 3 | 0.00 | 0.01 | 0.01 | 0.38 | 0.00 | 0.38 |
| 3 / 3' | 3 | 0.00 | 0.00 | 0.00 | 0.51 | 0.00 | 0.51 |
| 4 / 4' | 2 | 0.06 | 0.13 | 0.07 | 6.64 | 0.05 | 6.59 |
| 4 / 4' | 2 | 0.00 | 0.00 | 0.00 | 0.07 | 0.00 | 0.07 |
| 4 / 4' | 2 | 0.00 | 0.00 | 0.00 | 0.00 | 0.00 | 0.00 |
| 4 / 4' | 2 | 0.00 | 0.00 | 0.00 | 0.14 | 0.01 | 0.12 |
| 4 / 4' | 2 | 0.19 | 0.50 | 0.31 | 1.85 | 0.20 | 1.65 |
| 4 / 4' | 3 | 0.00 | 0.00 | 0.00 | 0.22 | 0.02 | 0.20 |
| 4 / 4' | 3 | 0.00 | 0.00 | 0.00 | 0.00 | 0.00 | 0.00 |
| 4 / 4' | 3 | 0.00 | 0.16 | 0.16 | 5.39 | 0.00 | 5.39 |
| 4 / 4' | 3 | 0.00 | 0.00 | 0.00 | 2.80 | 0.01 | 2.79 |
| 4 / 4' | 3 | 0.00 | 0.00 | 0.00 | 0.12 | 0.00 | 0.12 |

**Supplementary Table 3. Details of the File Preview library.** Four files are encoded and used in the Preview experiments. This table shows a description of the image, the size of the file in bytes, the number of strands per partition, and the fraction contained in each partition (% bytes and % strands), the forward and reverse primer binding sequence, their Hamming distance with respect to the 0 HD primers, the percent of file accessed including that partition (0 HD strands are accessed with 2 HD which are accessed with 4 HD which are accessed with 6 HD), the recognition and cut site for the restriction enzymes used for analysis, and the flanking primer sequences for the initial strand amplification when the library was received.

| **Wuflab Logo** | **Bytes** | **Percent of bytes stored** | **Strands** | **Percent of strands stored** | **Forward primer sequence** | **Reverse primer sequence** | **HD from primers** | **Percent of file accessed** | **Restriction site** | **Forward flanking primer** | **Reverse flanking primer** |
|---|---|---|---|---|---|---|---|---|---|---|---|
| 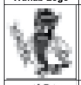 | 707 | 2.77% | 183 | 4.42% | **CAGGTACGCAGTTAGCACTC** | **CGTGGCAATATGACTACGGA** | 0 | 4.42% | GGTACC | CAGGAGAATGCCTTCCTAGG | CCTCGGTTCTTCTTGACCAG |
| 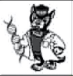 | 1763 | 6.91% | 338 | 8.16% | CATGTTCGCAGTTAGCACTC | CGTGGCTATATGACTACGCA | 2 | 12.57% | CCTGCAGG | AGGCTGGAGGTCCAATCTTG | ATTCTGGCCACTTCCTGAAG |
| 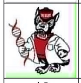 | 3462 | 13.58% | 597 | 14.41% | CCGATACGTAGTTAGCGCTC | CGGAGAAATATGACGACGGA | 4 | 26.98% | GCGGCCGC | AACTAAACGGAGGCCAACAG | TTTGTCCAGGAGCCTTTGAG |
| 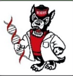 | 19565 | 76.73% | 3026 | 73.02% | TTATTACGCGGTGAGCACTC | CATAGCAATAAGGCTCCGGT | 6 | 100.00% | GTTTAAAC | CGTGGATTCAATTCGGAACG | TTGTTCGCCGAACTGGTTAG |
| **Wright Glider 1** | **Bytes** | **Percent of bytes stored** | **Strands** | **Percent of strands stored** | **Forward primer sequence** | **Reverse primer sequence** | **HD from primers** | **Percent of file accessed** | **Restriction site** | **Forward flanking primer** | **Reverse flanking primer** |
| 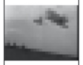 | 726 | 2.60% | 184 | 4.18% | **CAGGTACGCAGTTAGCACTC** | **CGTGGCAATATGACTACGGA** | 0 | 4.18% | GGTACC | CTGCCAACCTCGGATAACCG | GAACCGAACGGCCACAATAG |
| 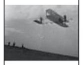 | 2455 | 8.78% | 415 | 9.42% | CCGATACGTAGTTAGCGCTC | CGGAGAAATATGACGACGGA | 4 | 13.60% | GCGGCCGC | AGGTCGGACAACGCCTTAAG | TCCACCAACGAACATTTACG |
| 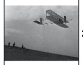 | 24785 | 88.63% | 3806 | 86.40% | TTATTACGCGGTGAGCACTC | CATAGCAATAAGGCTCCGGT | 6 | 100.00% | GTTTAAAC | CGGCACCAACGAAAGAATCG | CAACGAAGGTCCGTCCTTAG |
| **Wright Glider 2** | **Bytes** | **Percent of bytes stored** | **Strands** | **Percent of strands stored** | **Forward primer sequence** | **Reverse primer sequence** | **HD from primers** | **Percent of file accessed** | **Restriction site** | **Forward flanking primer** | **Reverse flanking primer** |
| 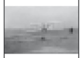 | 1187 | 3.84% | 236 | 4.84% | **CAGGTACGCAGTTAGCACTC** | **CGTGGCAATATGACTACGGA** | 0 | 4.84% | GGTACC | AGCCTGAACCGTTCTTCCTG | CGCGGAAGGAGGATTAACAG |
| 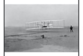 | 4433 | 14.36% | 705 | 14.47% | CATGTTCGCAGTTAGCACTC | CGTGGCTATATGACTACGCA | 2 | 19.31% | CCTGCAGG | TACCAACATTGCCGCAACTG | CGACCAACAAGGTTCTTACG |
| 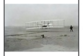 | 4437 | 14.37% | 705 | 14.47% | CAGGTAAGTAGCCAGCACTC | CAGGGCAATATGAGAACGGA | 4 | 33.78% | GCGGCCGC | CAACTTGTCCTCCATAAGCG | ACTTAAGCCAGGTTGATTCG |
| 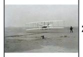 | 20822 | 67.43% | 3226 | 66.22% | CTGGTATGCCCTTAACACCC | CGTGGGGCTATGACTATGTC | 6 | 100.00% | GTTTAAAC | AATGTTCCTGTTGGCGGTTG | ACAATCCTAAGTCCGGTAGG |
| **Earth** | **Bytes** | **Percent of bytes stored** | **Strands** | **Percent of strands stored** | **Forward primer sequence** | **Reverse primer sequence** | **HD from primers** | **Percent of file accessed** | **Restriction site** | **Forward flanking primer** | **Reverse flanking primer** |
| 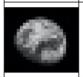 | 696 | 2.56% | 181 | 4.19% | **CAGGTACGCAGTTAGCACTC** | **CGTGGCAATATGACTACGGA** | 0 | 4.19% | GGTACC | ACCAACTAACGGCTTCGTTG | GTAACCATCCGGAGGAAGAG |
| 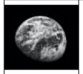 | 1950 | 7.17% | 359 | 8.31% | CAGGTAAGTAGCCAGCACTC | CAGGGCAATATGAGAACGGA | 4 | 12.50% | GCGGCCGC | GTTCTTGGCTCCAGGTAAGG | ACCGGTCAATTACAACGAAG |
| 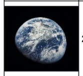 | 24555 | 90.27% | 3781 | 87.50% | CTGGTATGCCCTTAACACCC | CGTGGGGCTATGACTATGTC | 6 | 100.00% | GTTTAAAC | ACGGCGAAGGACAATTACGG | GTTAACACCGTGGCAACCAG |

**Supplementary Table 4. Wuflab logo and Wright Glider 2 files are partitioned into four parts.** The scans in each partition are described in each row.

| Partition | JPEG Progressive Encoding Components per Partition | Preview Description |
|---|---|---|
| 1 | JFIF header, Q0, SOF, Y[0] | **Preview.** JFIF is the JPEG header. Q0 is a quantization matrix. SOF marks beginning of scans. Y[0] provides a grayscale Preview. |
| 2 | Y[1:5] | **Intermediate Preview**. Improves resolution of grayscale. |
| 3 | Cb[0] ,Cr[0], Y[6:10], Y[11:15] | **Intermediate Preview**. Adds color and improved grayscale resolution. |
| 4 | Y[16:20], Y[21:25], Y[26:30], Y[31:35], Y[36:40],Y[41:45], Y[46:50], Y[51:55], Y[56:60], Y[61:63], Cc[1:5], Cr[1:5],CB[6:10], Cr[6:10], Cb[11:15], Cr[11:15], Cb[16:20], Cr[16:20], Cb[21:25], Cr[21:25], Cb[26:30], Cr[26:30], Cb[31:35], Cr[31:35], Cb[36:40], Cr[36:40] ,Cb[41:45], Cr[41:45], Cb[46:50], Cr[46:50], Cb[51:55], Cr[51:55], Cb[56:60], Cr[56:60], Cb[61:63], Cr[61:63], EOI | **Full Image.** Adds remaining components for full image reconstruction. EOI marks the end of the image. |

**Supplementary Table 5. Wright Glider 1 and Earth files are partitioned into three parts.** The scans in each partition are described in each row.

| Partition | Progressive Encoding Components per Partition | Preview Description |
|---|---|---|
| 1 | JFIF header, Q0, SOF, Y[0] | **Preview.** JFIF is the JPEG header. Q0 is a quantization matrix. SOF marks beginning of scans. |
| 2 | Y[1:5] | **Intermediate Preview**. Improves resolution of grayscale. |
| 3 | Cb[0], Cr[0], Y[6:10], Y[11:15], Y[16:20], Y[21:25], Y[26:30], Y[31:35], Y[36:40],Y[41:45], Y[46:50], Y[51:55], Y[56:60], Y[61:63], Cb[1:5], Cr[1:5],Cb[6:10], Cr[6:10], Cb[11:15], Cr[11:15], Cb[16:20], Cr[16:20], Cb[21:25], Cr[21:25], Cb[26:30], Cr[26:30], Cb[31:35], Cr[31:35], Cb[36:40], Cr[36:40] ,Cb[41:45], Cr[41:45], Cb[46:50], Cr[46:50], Cb[51:55], Cr[51:55], Cb[56:60], Cr[56:60], Cb[61:63], Cr[61:63], EOI | **Full Image.** Adds remaining components for full image reconstruction. EOI marks end of the image. |
